# Evaluating a Novel Regional Technique: Serratus Posterior Superior Intercostal Plane Block Reduces Opioid Consumption and Pain Scores after Breast-conserving Surgery: A Randomized Controlled Trial

**DOI:** 10.4274/TJAR.2026.252202

**Published:** 2026-06-26

**Authors:** Bahadır Çiftçi, Burak Ömür, Birzat Emre Gölboyu, Selçuk Alver, Pelin Basim, Tumay Uludağ Yanaral, Bayram Ufuk Sakul

**Affiliations:** 1 İstanbul Medipol University Faculty of Medicine, Department of Anaesthesiology and Reanimation, İstanbul, Türkiye; 2 İstanbul Medipol University Faculty of Medicine, Department of Anatomy, İstanbul, Türkiye; 3 İzmir Katip Çelebi University Faculty of Medicine, Department of Anaesthesiology and Reanimation, İzmir, Türkiye; 4 Biruni University Faculty of Medicine Hospital, Department of Anaesthesiology and Reanimation, İstanbul, Türkiye; 5 İstanbul Medipol University Faculty of Medicine, Department of General Surgery, İstanbul, Türkiye

**Keywords:** Breast surgery, chest wall blocks, pain management, regional anaesthesia, serratus posterior superior intercostal plane block, ultrasound

## Abstract

**Objective:**

Serratus posterior superior intercostal plane block (SPSIPB) provides thoracic analgesia. Our objective was to assess the analgesic effectiveness of SPSIPB in reducing pain scores and opioid consumption in patients undergoing breast-conserving surgery (BCS) with axillary dissection or sentinel lymph node biopsy.

**Methods:**

Participants were individuals aged 18-65 years with American Society of Anesthesiologists physical status I-II who were scheduled for elective BCS under general anaesthesia. Participants were randomly assigned to Group SPSIPB (n = 30) or Group Control (n = 30); the control group received local infiltration anaesthesia. A total of 30 milliliters of 0.25% bupivacaine was during the SPSIPB procedure. The primary outcome of the study was the numerical rating scale (NRS) score at 1 hour postoperatively. Secondary outcomes included 24-hour opioid consumption, need for rescue analgesia, and adverse effects.

**Results:**

During the first 24 hours after surgery, the median static and dynamic NRS scores were lower in the SPSIPB group than in the control group (*P* < 0.005). Fewer patients in the SPSIPB group required rescue analgesia than in the control group (3 vs. 26 patients, *P*=0.001), and opioid consumption was lower in the SPSIPB group (*P*=0.001). The incidence of adverse effects was significantly lower in the SPSIPB group (*P* < 0.005).

**Conclusion:**

Opioid consumption and pain scores in the SPSIPB group were significantly lower compared with those in the control group. SPSIPB provides effective analgesia and reduces opioid requirements, offering a valuable opioid-sparing alternative for anaesthesia in breast surgery.

**Main Points**

•  Serratus posterior superior intercostal plane block (SPSIPB) is a novel regional anaesthesia method that provides thoracic analgesia.

•  Case reports and limited studies report that SPSIPB provides effective analgesia for breast surgery.

•  This is a prospective, randomized study of the efficacy of SPSIPB for breast surgery, reported in the literature.

•  Our results indicate that SPSIPB provides effective analgesic management in patients who underwent breast surgery.

## Introduction

Breast-conserving surgery (BCS), commonly called lumpectomy, is a common procedure for treating breast cancer, one of the most prevalent cancers affecting women worldwide.^1^ Breast cancer accounts for nearly one in three new cancer diagnoses among women, underscoring the need for effective, patient-centered surgical options. BCS involves removing the cancerous tumor while preserving as much of the breast as possible, often resulting in favorable cosmetic outcomes and psychological benefits.^1^ However, a significant number of patients report persistent pain following breast surgery, ranging from mild discomfort to chronic pain.^2^ This pain arises from multiple mechanisms, including nerve damage or irritation during surgery, tissue inflammation, and scarring, all of which can cause long-term discomfort. Other sources of pain after breast surgery include axillary procedures, such as axillary dissection and sentinel lymph node biopsy.^3^ In the acute postoperative phase, patients who have undergone breast surgery, particularly axillary surgery, experience severe pain. Addressing these pain mechanisms early, through individualized pain-management strategies, is essential to improving quality of life in patients who have undergone BCS.^1, 2, 3^

The PROSPECT guidelines propose the use of regional anaesthetic procedures. More recently, the 2023 evidence-based guideline on the prevention and management of perioperative pain for breast cancer surgical patients emphasizes multimodal analgesia to minimize opioid use.

Effective pain management is crucial for patients undergoing breast surgery, as it not only improves recovery but also reduces the risk of chronic pain.^2, 3^The PROSPECT guidelines propose using regional anaesthetic procedures.^2^ More recently, the 2023 Evidence-based Guideline on the Prevention and Management of Perioperative Pain for Breast Cancer Surgical Patients emphasizes multimodal analgesia to minimize opioid use.^4^ Regional analgesia techniques have become popular for managing postoperative pain after breast surgery, offering targeted pain relief while minimizing the need for systemic opioids.^3^ With the growing use of ultrasound (US) in daily anaesthesia practice, several fascial plane block techniques are used to provide analgesia for chest wall surgeries. By using these regional analgesia techniques, patients experience not only effective pain relief but also a reduced need for opioids, which can lower the risk of side effects and enhance post-operative recovery.^3^ In recent years, novel techniques have emerged to address the limitations and inconsistencies in prior methods. The serratus posterior superior intercostal plane block (SPSIPB), introduced by Tulgar et al.^5^ in 2023, represents a recent advancement in regional anaesthesia. In their study, which included one cadaver and five patients, they demonstrated that SPSIPB spreads from C7 to T7, effectively involving the intercostal nerves in the cadaver and providing a broad hemithoracic sensory blockade in the patients.^5^ Existing case reports support that it is efficacious for various breast operations.^6, 7, 8, 9, 10, 11, 12^ However, to our knowledge, no randomized clinical trials have investigated its use specifically in breast surgery. Therefore, we designed this study to evaluate the effectiveness of SPSIPB in this context, hypothesizing that it would offer superior analgesia compared to a control group receiving local infiltration. This study aims to compare SPSIPB and local infiltration with respect to pain scores, opioid requirements, and adverse events.

## Methods

### Study Design

This single-center, prospective, randomized study received approval from the Ethics and Research Committee of the İstanbul Medipol University Non-interventional Clinical Research Ethics Board (approval no.: 365, date: 13.04.2023). Following ethical approval, the study protocol was registered on ClinicalTrials.gov (NCT05972083). Participants were patients aged 18 to 65 with ASA classification I-II who were scheduled to undergo elective BCS with either axillary dissection or sentinel lymph node biopsy. Exclusion criteria included refusal to participate, posterior thoracic wall infection, coagulation disorders, pregnancy, inability to score pain, and a history of allergic reactions to local anaesthetics or study drugs. The study, conducted at Medipol Mega University Hospital, spanned from August 2023 to October 2024, and all participants provided written informed consent.

### Grouping, Blinding, and Randomization

Prior to surgery, participants were randomized in a 1:1 ratio to the SPSIPB group (n = 30) or the control group (n = 30). The Research Randomizer computer program, which created a randomization table and assigned each patient an ID, was used to oversee the randomization process. The group allocations were concealed from the patients and the pain nurse-anaesthetist who evaluated the surgical outcomes. To maintain consistency, all blocks were performed by an anaesthesiologist skilled in regional anaesthesia.

### General Anaesthesia Management and Surgical Technique

The clinic’s standard anaesthesia protocol was used for induction and maintenance of general anaesthesia, with a multimodal analgesic regimen of 400 mg ibuprofen and 100 mg tramadol administered intravenously (IV) 20 minutes before surgery. Additionally, 4 mg IV ondansetron was administered for prophylaxis of postoperative nausea and vomiting. All surgeries, including BCS with axillary dissection or sentinel lymph node biopsy, were performed by the same surgical team following a standardized technique.

### SPSIPB Procedure

**Figure 1:**
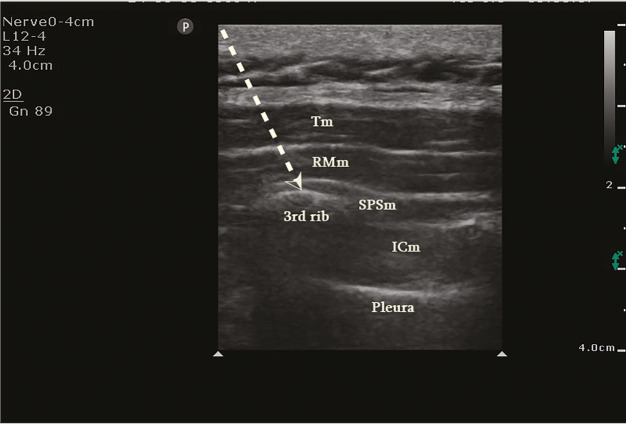
Sonographic visualization of SPSIPB. The Trapezius, rhomboid major, serratus posterior superior, the third rib, and the pleura are visualized. The arrow indicates the needle trajectory. The tip of the arrow is located between SPSm and the third rib. Tm, trapezius muscle; RMm, rhomboideus major muscle; SPSm, serratus posterior superior muscle; ICm, intercostal muscle; SPSIPB, serratus posterior superior intercostal plane block.

Patients were placed in the lateral decubitus position (surgical side up) after completion of the sterile SPSIPB procedure and before extubation. The scapular spine serves as a crucial anatomical landmark for the SPSIPB.^5^ After the manual palpation of the scapular spine, a high-frequency transducer (4-12 MHz) was placed sagittally on the scapula. The scapular spine was identified using US, and the transducer was moved toward the upper medial border of the scapula. The third rib was visualized adjacent to the medial border, with a slight oblique angulation applied to the transducer for optimal imaging.^4, 8^ The trapezius muscle, rhomboid major muscle, serratus posterior superior muscle (SPSM), third rib, and pleura were visualized. A 22G, 80-mm block needle (Stimuplex^®^ Ultra 360^®^, B. Braun, Melsungen, Germany) was directed between the SPSM and the third rib. The block site was confirmed by injecting 5 mL of isotonic solution between the SPSM and the rib. Subsequently, 30 mL of 0.25% bupivacaine was administered into this plane (Figure 1).

In the control group, the surgical team performed wound infiltration along the incision line and into breast tissue. Additional infiltration was applied to the axillary fossa following dissection (a total of 30 ml of 0.25% bupivacaine). Surgical drains were placed in patients.

### Postoperative Analgesia Regimen and Outcomes

For postoperative pain management, 400 mg of intravenous ibuprofen was prescribed every 8 hours as part of the routine analgesic protocol. Pain was measured using the numerical rating scale (NRS), with 0 indicating no pain and 10 indicating the worst imaginable pain. In the post-anaesthesia care unit and at 2, 4, 8, 16, and 24 hours after surgery, both static and dynamic NRS scores were recorded. As a rescue analgesic, 0.5 mg kg^-1^ intravenous meperidine was administered to patients with an NRS score of 4 or higher.

The NRS score one hour after surgery was the study’s main endpoint. Use of meperidine, a rescue opioid analgesic, and the prevalence of adverse effects such as nausea, vomiting, and itching were secondary outcomes.

### Sample Size

The G*Power program (version 3.1.9) was used to determine the study’s sample size. The comparison of the first-hour NRS scores was the primary objective. In a preliminary analysis that included eight patients in each group, first-hour postoperative NRS score was 1 [standard deviation (SD) 0.55] in the SPSIPB block group and 3 (SD 2.25) in the control group. A minimum of 26 patients per group was needed to achieve 95% power, assuming an α error of 0.05 and a β error of 0.01. To account for potential dropouts, we decided to include at least 30 patients in each group.

### Statistical Analysis

The shapes of the distributions of the study variables were evaluated using the Shapiro-Wilk test to assess normality and detect skewness. An independent samples t-test was used for group comparisons, and values for normally distributed data were displayed as the mean ± SD. The results were presented as median and interquartile range for continuous data that were not normally distributed. Group differences were then examined using the Mann-Whitney U test. Statistical significance was defined as *P* < 0.05. SPSS (version 25, SPSS Inc., Chicago, IL, USA) was used for all analyses.

## Results

**Figure 2:**
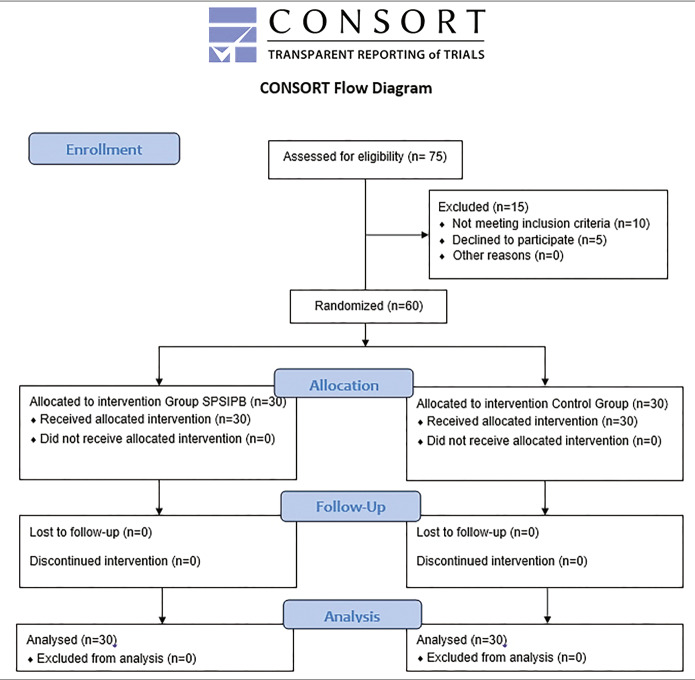
CONSORT flow diagram of the study.

The CONSORT flowchart (Figure 2) was used to track patient enrollment in this prospective, randomized study. Patient enrollment flow is detailed in Figure 2. Demographic data, surgery duration, and anaesthesia times were similar between groups (Table 1).

Table 2 presents a comparison of NRS scores at rest and during movement between groups. NRS scores were consistently lower in the SPSIPB group across all time points within the first 24 postoperative hours (*P*=0.001)

Data concerning the number of patients requiring rescue analgesia and opioid consumption (meperidine) are presented in Table 3. Patients in the control group required rescue analgesia at a significantly higher rate than those in the SPSIPB group (26 vs. 3 patients; *P*=0.001). Furthermore, the control group consumed significantly more opioids than the SPSIPB group [0 (0-0) vs. 60 (30-70); *P*=0.001]. The median value of 60 mg represents cumulative consumption of rescue analgesia over 24 hours, not a single dose.

The control group experienced a significantly higher incidence of adverse effects than the SPSIPB group: nausea, vomiting, and itching occurred in 18, 9, and 10 patients versus 2, 1, and 2 patients, respectively (*P* < 0.005) (Table 3). Our data show that the control group experienced pain; they received a cumulative rescue meperidine dose equal to twice the standard dose and, because meperidine commonly causes pruritus and vomiting, they experienced these side effects.

## Discussion

The results of the research indicate that SPSIPB is a successful and practical approach for managing pain in patients who underwent BCS. Compared to the control group, the SPSIPB group reported lower NRS scores both at rest and during movement throughout the 24-hour postoperative period, as well as reduced opioid consumption, reduced demand for rescue analgesics, and fewer patients requiring additional pain relief. Additionally, the incidence of nausea, vomiting, and itching was lower in the SPSIPB group. The significantly higher incidence of adverse effects, including nausea, vomiting, and pruritus, observed in the control group, appears to be strongly correlated with the increased consumption of rescue opioids. The limited analgesic coverage provided by local infiltration alone likely necessitated increased meperidine use, resulting in these dose-dependent side effects. In contrast, the SPSIPB group demonstrated a clear opioid-sparing benefit, which translated directly into a more favorable adverse effect profile.

Breast surgery is frequently associated with significant acute and chronic postoperative pain, which substantially compromises patient comfort, especially after procedures such as mastectomy and axillary lymph node dissection. Postoperative pain is prevalent, with approximately 50% of patients experiencing severe acute pain that can contribute to chronic pain. This level of discomfort can impede recovery, affecting breathing and delaying mobilization.^1, 2, 3^Consequently, regional anaesthesia techniques are frequently employed to optimize postoperative pain management in breast surgery patients.^3^ The PROSPECT guidelines propose using regional anaesthetic procedures to provide postoperative analgesia after breast surgery.^2^

Various regional anaesthesia techniques are available for pain management following breast surgery. As plane-block methods have evolved, newer interfascial plane blocks have been introduced to address the limitations of earlier techniques. For instance, the paravertebral block, while effective, carries a high risk of pneumothorax due to its proximity to the pleura.^3^ The erector spinae plane block (ESPB), introduced in 2016, is another option for patients undergoing breast surgery.^13, 14^ However, its mechanism of action remains controversial, with imaging and cadaveric studies demonstrating inconsistent patterns of spread.^14, 15, 16, 17, 18^ Additionally, dermatome analyses have revealed that ESPB performed at the same level can produce varying sensory block levels among different individuals.^19^ Another technique, the rhomboid intercostal block (RIB), has limited axillary spread and may not consistently cover areas beyond the T3 dermatome.^5, 20^ Given these limitations, SPSIPB offers a promising alternative for patients undergoing breast surgery and axillary dissection.

The SPSIPB technique, first defined by Tulgar et al.^5^ in 2023, involves the injection of a local anaesthetic between the SPSM and the third rib. The SPSM is a thin, membranous, periscapular muscle with an oblique course, extending from the C7-T3 vertebral levels to the lateral aspects of the second and fifth ribs. This unique structure allows the anaesthetic to spread widely when injected into the SPSM’s deep fascia.^21^ Cadaver studies have reported an extensive spread from C7 to T7, involving the intercostal nerves and potentially reaching the dorsal rami of the spinal nerves.^5^ In a case series by Ciftci et al.,^9^ SPSIPB was applied to three breast surgery patients; all exhibited low pain scores and required no additional analgesics. Dermatomal analysis in these cases demonstrated sensory blockade from C3 to T10, including the axilla. Our study aligns with these findings, with patients showing consistently low pain scores and reduced opioid use compared to the control group. In summary, SPSIPB may offer a practical, safe, and effective pain management option for breast surgery.

A notable advantage of SPSIPB for breast surgery is its distance from the surgical field, meaning that the block’s effectiveness is less likely to be compromised by surgical tissue trauma. We performed SPSIPB by positioning the patient in the lateral decubitus position before extubation. Unlike ESPB, which may be challenging to perform in the lateral position due to the depth of the transverse process and difficulties in probe handling, SPSIPB is more straightforward. The rib, being more superficial and lateral, facilitates visualization, and placing the probe medial to the scapula improves grip and control. While case reports suggest that SPSIPB provides effective analgesia in various surgeries, randomized controlled trials remain limited. Avci et al.^22^ compared SPSIPB with a control group in thoracoscopic surgery and reported that SPSIPB provided effective analgesia. In another clinical study by Köksal et al.,^23^ the authors compared SPSIPB with the control group and reported that SPSIPB provided effective postoperative analgesia in patients who underwent breast cancer surgery. Unlike our study, Köksal et al.^23^ reported effective analgesia with 20 mL of local anaesthetic. In our study, we used 30 mL, hypothesizing that a larger volume might improve interfascial spread. While both studies report success, future dose-finding studies are needed to determine the optimal volume-to-efficacy ratio for SPSIPB. Our study is the second clinical investigation of SPSIPB to focus on its application to breast surgery.

Yu et al.^24^ compared RIB, serratus anterior plane block (SAPB), and paravertebral block with respect to the quality of recovery after breast cancer surgery. They reported that RIB and the paravertebral block provided similar analgesic effects for breast cancer surgery. However, according to their results, the analgesic effect of the SAPB was inferior to that of the RIB and the paravertebral block. They emphasized that RIB may be one of the best alternatives to the paravertebral block among fascial plane blocks. Similarly, Altıparmak et al.^25^ reported that RIB provided effective analgesic management compared with the control group after breast surgery. However, consistent with our results, SPSIPB, unlike the RIB (which focuses on T2-T9), facilitates cranial spread from C7 to T7, potentially providing superior coverage for the high axillary pain often associated with BCS involving lymph node dissection. Our results using SPSIPB suggest that it may offer a distinct advantage in more consistently targeting the dorsal rami and lateral cutaneous branches than SAPB, which is primarily an anterolateral block. Abdella et al.^26^ evaluated the analgesic efficacy and the spread of varying volumes of local anaesthetic in the ESPB. They reported that doubling the injectate volume enhances the craniocaudal spread and may be useful for surgeries involving multiple dermatomes. However, according to their results, larger volume has no effect on analgesic efficacy or patient satisfaction because there is no further spread to the paravertebral, epidural spaces, or spinal nerve rami.

### Study Limitations

Our study has some limitations. Although sensory blockade assessments are generally recommended to evaluate the effectiveness of plane blocks, we did not perform a dermatome analysis. We used 30 milliliters of local anaesthetic because plane-block effectiveness varies with volume; different outcomes may therefore occur with other volumes. Furthermore, we had a small sample size. Larger-scale clinical trials are required to demonstrate more conclusively the effectiveness of SPSIPB. Although local infiltration is a standard analgesic method, the control group in our study demonstrated a high requirement for rescue analgesia. This suggests that in BCS with axillary involvement, infiltration alone may be insufficient to address the complex pain mechanisms, or that the specific infiltration technique used may have provided limited coverage compared to the fascial plane spread of SPSIPB. Meperidine was used as the rescue analgesic in accordance with our institution’s standard postoperative protocol during the study period, though we acknowledge that other opioids are more commonly used internationally.

## Conclusion

SPSIPB offers effective analgesia for patients undergoing BCS with axillary dissection, significantly lowering pain scores and reducing opioid requirements.

## Ethics

**Ethics Committee Approval:** This single-center, prospective, randomized study received approval from the Ethics and Research Committee of the İstanbul Medipol University Clinical Research Ethics Board (approval no.: 365, date: 13.04.2023).

**Informed Consent:** All participants were informed about the study, and their written consent was obtained.

## Figures and Tables

**Table 1. Comparison of Demographic Data and Duration Times of Surgery and Anaesthesia Between Groups:** 

-	**Group SPSIPB (n = 30)**	**Group control (n = 30)**	***P***
Age	53 (42-61)	49 (42-54)	*0,307
ASA (I/II)	7/23	7/23	†1
Height (cm)	162 (157-168)	162 (158-168)	*0.773
Weight (kg)	72 (65-80)	68 (35-76)	*0.251
Duration of surgery (min)	80 (71-93)	86 (80-98)	*0.125
Duration of anaesthesia (min)	95 (80-108)	101 (95-116)	*0.092

Values are expressed median (percentiles 25-75) or number, *: *P* value is obtained with Mann-Whitney U test, †: *P* value is obtained with Pearson’s χ2 test (n), *P* values were italicized and values that are written in bold represent statistical significance

ASA, American Society of Anesthesiologist; m, male; f, female; cm, centimeter; kg, kilogram; min, minutes; SPSIPB, Serratus posterior superior intercostal plane block

**Table 2. Comparisons of Static and Dynamic NRS Assessment Between Groups:** 

-	**Group SPSIPB (n = 30)**	**Group control (n = 30)**	***P***
**At rest**			
1^st^ hour	0 (0-1)	2 (2-3)	**0.001**
2^nd ^hour	0 (0-1)	2 (2-3)	**0.001**
4^th^ hour	0 (0-0)	2 (1-3)	**0.001**
8^th^ hour	0 (0-0)	2 (1-3)	**0.001**
16^th^ hour	0 (0-0)	2 (1-2)	**0.001**
24^th^ hour	0 (0-0)	1 (1-2)	**0.001**
**On movement**			
1^st^ hour	0 (0-2)	4 (3-5)	**0.001**
2^nd^ hour	1 (0-2)	4 (3-4)	**0.001**
4^th ^hour	0 (0-1)	3 (3-3)	**0.001**
8^th ^hour	0 (0-1)	3 (2-3)	**0.001**
16^th^ hour	0 (0-0)	2 (2-3)	**0.001**
24^th^ hour	0 (0-0)	2 (2-3)	**0.001**

Data are expressed as median (percentiles 25-75), *P* value is obtained with Mann-Whitney U test median (percentiles 25-75), *P* values were italicized and values that are written in bold represent statistical significance

NRS, numeretic rating pain scale; SPSIPB, serratus posterior superior intercostal plane block

**Table 3. The Comparison of Opioid (Meperidine) Consumptions, the use of Rescue Analgesia and the Incidence of Side Effects Between Groups:** 

-	**Group SPSIPB (n = 30)**	**Group control (n = 30)**	***P***
**Rescue analgesia **(the number of the patients that used rescue analgesia) **(Y/N)**	3/27	26/4	**0.001**
**Rescue dose (mg)**	0 (0-0)	60 (30-70)	**0.001**
**Nausea (Y/N)**	2/28	18/12	**0.001**
**Vomiting (Y/N)**	1/29	9/21	**0.006**
**Itching (Y/N)**	2/29	10/20	**0.001**

*P* value is obtained with Pearson’s χ2 test (n), Data are expressed as median

*: *P* value is obtained with Mann-Whitney U test median (percentiles 25-75), *P* values were italicized and values that are written in bold represent statistical significance
